# Knowledge Discovery from Posts in Online Health Communities Using Unified Medical Language System

**DOI:** 10.3390/ijerph15061291

**Published:** 2018-06-19

**Authors:** Donghua Chen, Runtong Zhang, Kecheng Liu, Lei Hou

**Affiliations:** 1 Department of Information Management, School of Economics and Management, Beijing Jiaotong University, Beijing 100044, China; 15113181@bjtu.edu.cn; 2 Henley Business School, University of Reading, Reading RG6 6UD, UK; k.liu@reading.ac.uk (K.L.); l.hou@pgr.reading.ac.uk (L.H.)

**Keywords:** online posts, online health communities, knowledge discovery, Unified Medical Language System, text mining

## Abstract

Patient-reported posts in Online Health Communities (OHCs) contain various valuable information that can help establish knowledge-based online support for online patients. However, utilizing these reports to improve online patient services in the absence of appropriate medical and healthcare expert knowledge is difficult. Thus, we propose a comprehensive knowledge discovery method that is based on the Unified Medical Language System for the analysis of narrative posts in OHCs. First, we propose a domain-knowledge support framework for OHCs to provide a basis for post analysis. Second, we develop a Knowledge-Involved Topic Modeling (KI-TM) method to extract and expand explicit knowledge within the text. We propose four metrics, namely, explicit knowledge rate, latent knowledge rate, knowledge correlation rate, and perplexity, for the evaluation of the KI-TM method. Our experimental results indicate that our proposed method outperforms existing methods in terms of providing knowledge support. Our method enhances knowledge support for online patients and can help develop intelligent OHCs in the future.

## 1. Introduction

Online Health Communities (OHCs) help patients exchange experiences through posts [1] and are particularly useful for patients with chronic conditions to help manage their health [2]. Posts in OHCs often contain massive textual descriptions, including information on drug use, patient conditions, and patient-centered events [3]. Therefore, with massive effective medical and healthcare information, OHCs can be used to predict crucial health events that online patients with life-changing illnesses may experience [4]. A knowledge-based OHC can also help healthcare providers, online health information entrepreneurs, and developers make intelligent choices for patients and their caregivers [5,6]. The inclusion of effective recommendation mechanisms in healthcare-related social platforms can promote the involvement of users in the management of their personal health. The maintenance of an effective OHC requires effective community management to provide additional online support to patients. A patient’s needs can be predicted by using the emotional, social, and technical contexts of their online posts [7]. However, information systems in current OHCs continue to encounter difficulties in the automatic extraction of meaningful relationships from various types of medical information in posts because health-related narrative posts are highly complex [8,9,10,11]. Thus, effective methods for the extraction of useful relationships among medical terms retrieved from texts should be investigated.

The Unified Medical Language System (UMLS) Metathesaurus is provided by the National Library of Medicine. The UMLS has been used as a complete knowledge source in the medical and healthcare field. We posit that we can improve the capabilities of OHCs by introducing the massive medical concepts and relationships contained by the UMLS to the extraction of useful medical knowledge from posts in OHCs. The Systematized Nomenclature of Medicine-Clinical Terms (SNOMED CT) is the most important terminology set in the UMLS. It is maintained by the International Health Terminology Standards Development Organization and defines numerous concepts and concept relationships in the medical field. In recent years, the SNOMED CT has been widely used and recommended as a reference for the exchange of terminologies in medical and clinical informatics [12,13]. However, a fully intelligent online community for patients with chronic conditions cannot be established by merely relying on the presence of medical professionals in OHCs [14]. Many practical systems facilitated by machine-learning methods have improved medical knowledge discovery from medical texts on the basis of the UMLS Metathesaurus [15,16]. The UMLS can support efficient transitive queries and concept relationship studies on OHCs [17]. Despite the complexity of clinical data analysis, the UMLS Metathesaurus and SNOMED CT remain as effective biomedical knowledge sources for the information extraction of problems, procedures, medications, and clinical results and are consequently widely used in web-based contexts. For example, medical knowledge has been introduced to a web-based context to facilitate medical knowledge discovery through social media practices [18]. Moreover, the multidisciplinary and complex biomedical information provided by these knowledge sources are necessary to support online patients with chronic conditions. However, post utilization remains challenging when domain knowledge is integrated into OHCs. Although many researchers have developed and refined SNOMED CT for clinical uses, additional studies on its online use should be conducted [19].

The rest of this paper describes a comprehensive method based on the UMLS Metathesaurus for the extraction of medical concepts and discovery of concept relationships for knowledge-based services in OHCs. In addition, this paper describes the derivation of metrics necessary for evaluating the performance of the proposed method. Here, we first describe and explore the framework of domain knowledge support in OHCs. Then, we propose a method for the discovery of medical knowledge from posts. Finally, we evaluate our work and provide recommendations for improving the services of OHCs.

## 2. Materials and Methods

### 2.1. Framework of Domain Knowledge Support

**Figure 1:**
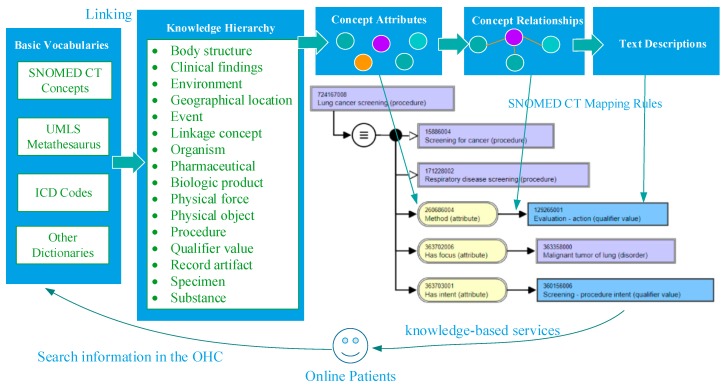
Overview of knowledge sources in the medical and healthcare field.

To achieve the objective of our study, we consider integrating Domain Knowledge Support (DKS) into the information systems of OHCs. In the medical field, DKS is essential for facilitating the analysis of online posts [20]. We use source vocabularies from the UMLS Metathesaurus to establish the framework shown in Figure 1. As mentioned, UMLS Metathesaurus and SNOMED CT are effective biomedical knowledge sources for the information extraction of the posts. The knowledge sources contain not only definitions of concept, but they also include synonyms and semantic relationships that can be used for knowledge discovery. The DKS integrates the knowledge sources and reorganizes them to be suitable for knowledge discovery from posts in the OHCs. Then we establish the Domain-Knowledge Support Framework (DKSF) to support our method.

The knowledge sources used in the DKSF shown in Figure 1 include Basic Vocabulary (*BV*), Knowledge Hierarchy (*KH*), Concept Attributes (*CA*), Concept Relationships (*CR*), and Textual Definitions (*TD*). Generally, a concept in DKSF is defined as follows:

(1) concept=(code,{CR},{CA},{TD}).

In (1), *code* represents a unique identification (ID) in *BV*. Then, we define a *CR* among concepts as follows:

(2) $$CR=((s,code_{s}),\{r\},(t,code_{t})).$$

In (2), *s* represents the source concept, and *t* represents the target concept in a relationship set {*r*}. Meanwhile, *code* of *s* and *code* of *t* represent unique ID in *BV* for the first and second concepts, respectively. On the basis of (1) and (2), we define *BV* as follows:

(3) BV={{(KB,{CR})}}.

On the basis of (3), we define a *KH* as follows:

(4) KH={CR|∀r=IS_A}.

We reorganize the knowledge extracted from the UMLS Metathesaurus by using (1) to (4) and provide a framework for the analysis of posts in an OHC. The element-mapping relationships between the UMLS Metathesaurus and our DKSF are presented in Table 1. The use of knowledge relationships in the medical field facilitates the development of OHCs [21]. The detailed process of analyzing online posts is as follows: First, terms extracted from online posts are mapped to concepts in BV. Second, the concepts are linked to a specific *KH*. Third, relevant medical concepts and relationships are extracted from UMLS-based knowledge sources. Finally, relevant knowledge can be integrated in the decision-making process of online patients.

To analyze posts on the basis of DKSF, we transform the text from a post according to a structured model that supports entity recognition and entity relation discovery within the text. First, the model segments a text into a group of terms. Second, it determines the part-of-speech of each term within the text. Third, it determines domains for each term of the text. Finally, it reorganizes the text in accordance with the results obtained through previous steps. After analysis, the *text* is presented as

(5) $$text_{ws}=\{(term,pos,medical\_domain)\}.$$

**Figure 2:**
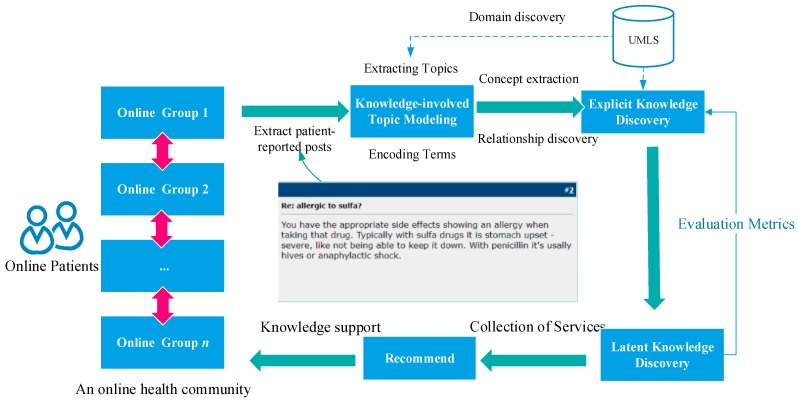
Overview of the domain-knowledge support framework in online health communities.

In (5), we encode terms within posts in a triple form (*term*, part-of-speech (*pos*), *medical_domain*). Therefore, the information contained by encoded text increases. The architecture of the OHC that uses DKS and based on DKSF is shown in Figure 2. The enhanced OHC finally provides knowledge-based support for online patients in different disease-based social support groups and a basis for research on the narrative experiences of online patients.

In OHCs, a patient-reported post is defined as

(6) $$online\_post=(\{background\},\{label\},\{topic\},caption,text_{ws},\{attribute\}).$$

In (6), an *online_post* provides not only narrative text but also other important information, such as background, label, topic, caption, and post attributes [22]. Here, we extract information for the analysis of posts by using novel knowledge-involved techniques. Accordingly, the method based on expanded medical knowledge within the text can be used to improve the services provided by OHCs [23]. Moreover, we propose evaluation metrics that are suitable for evaluating the knowledge extracted from posts. The proposed metrics are presented in the next section.

### 2.2. Method for Knowledge Discovery from Patient-Reported Posts

**Figure 3:**
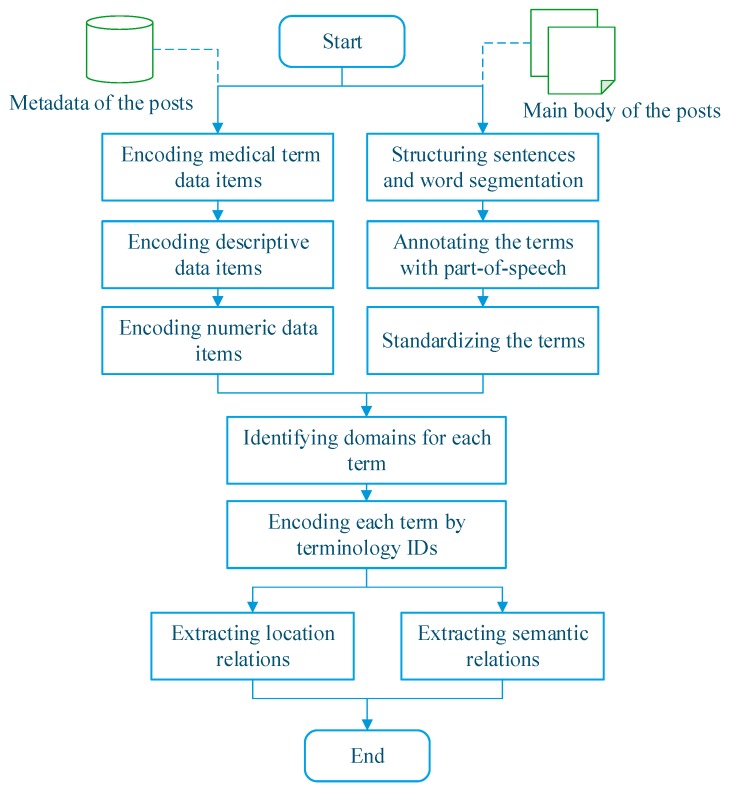
Flowchart of online-post analysis.

Here, we propose a comprehensive method based on DKS for knowledge discovery from patient-reported posts in OHCs to provide knowledge-based services. The knowledge sources used in the method are shown in Figure 1, and the DKS framework is shown in Figure 2. Figure 3 illustrates our analytical method. The four stages in this process include post classification, domain identification, term encoding, and relationship extraction among medical- and health-related concepts.

First, we cluster posts into a group of health-related topics. We classify a post in accordance with its topic under the assumption that each post with multiple labels has a topic related to a specific health-related concept with different backgrounds. For example, a post about cirrhotic ascites is classified by using a domain knowledge model related to liver disorders. Original posts are classified into predefined health-related topics used in knowledge models. The process shown in Figure 3 distinguishes medical text from a complete post into two types of data: metadata and narrative texts. Metadata text includes basic post information, such as post captions. Narrative text refers to the main body of a post. Several query generation methods, including topic modeling and key concept identification, have been proposed to convert long electronic health record notes to effective queries [24,25]. Algorithm 1 presents our Knowledge-Involved Topic Modeling (KI-TM) method, which is based on Latent Dirichlet Allocation (LDA), for processing the narrative text *source*. The techniques in the KI-TM method are *DomainDiscovery*, *TermEncoder*, and *LatentKnowledgeDisocovery*. KI-TM uses these techniques to establish a knowledge-involved LDA model for online posts.

Second, we use the *DomainDiscovery* algorithm in Algorithm 1 to identify the domains of terms extracted from posts. A domain for a medical term represents a type of medical meaning [26]. For example, the term “headache” belongs to the domain of patient condition. Medical domains, such as the descriptions of drug use and patient condition, are useful for the future implementation of knowledge reasoning. Here, we annotate the *pos* for each term and then determine each term’s domain on the basis of their *pos*. The determined domains are then mapped to the medical concepts in the *BV* of DKFS. The UMLS Metathesaurus is also composed of different terminology sets from numerous health-related domains. Such a composition contributes to identifying domains necessary for examining semantic relationships among concepts. A detailed algorithm for the labels of online posts is presented in Algorithm 2. When *m* = 1 in *labels*, the algorithm can be used for each term within the text where a label from an input is the term itself within the text.

**Algorithm 1** Knowledge-involved topic modeling method**Input**: *source*={(id_field, label_field, text_field)} **Output**: *KI-TM*(1)**Let***tokenizer* ← {(2)    SimpleEnglishTokenizer() ~>         // tokenize on space and punctuation(3)    CaseFolder() ~>               // lowercase everything(4)    WordsAndNumbersOnlyFilter() ~>     // ignore non-words and non-numbers(5)    MinimumLengthFilter(3)          // take terms with >=3 characters(6)    }(7)**Let***labels* ← {(8)    *source* ~>                    // read from the source(9)    Column(label_field) ~>             // take the column containing the text(10)    TokenizeWith(WhitespaceTokenizer()) ~>    // turns label field into an array(11)    ***DomainDiscovery*()**~>              // discover latent knowledge(12)    TermCounter() ~>                // collect label counts(13)    TermMinimumDocumentCountFilter(10)      // filter labels in < 10 docs(14)    }(15)**Let***text* ← {(16)    *source* ~>                   // read from the source file(17)    Column(text_field) ~>              // select column containing text(18)    TokenizeWith(*tokenizer*) ~>           // tokenize with tokenizer above(19)    TermCounter() ~>               // collect counts (needed below)(20)    TermMinimumDocumentCountFilter(4) ~>  // filter terms in <4 docs(21)    TermDynamicStopListFilter(30) ~>       // filter out 30 most common terms(22)    DocumentMinimumLengthFilter(5)     // take only docs with >=5 terms(23)    ***TermEncoder***()(24)    ***LatentKnowledgeDiscovery***()(25)    }(26)**Let***dataset* ← LabeledLDADataset(*text*, *labels*);(27)**Let***modelParams* ← LabeledLDAModelParams(dataset);(28)*KI-TM* ← TrainCVB0LabeledLDA(*modelParams*, *dataset*);(29)**return***KI-TM*

**Algorithm 2** Domain discovery algorithm**Input**: *labels*={*l*_1_, *l*_2_, …, *l_m_*} **Output**: domain labels of medical background
(1)**Let***results*←an empty list of domains {*l*,{*d*}} for each label in *labels* of a post(2)**Let***domains*←{*d*_1_, *d*_2_, …, *d_n_*} ⊆ medical knowledge in OHCs(3)**for each***l_i_* ∊ *labels* **do**(4)  **for each** *d_j_* ∊ *domains* **do**(5)     **if** *l_i_* ∊ *d_j_* **then**(6)      *results*[*l_i_*]←normalized *d_j_* in UMLS(7)     **end if**(8)   **end for**(9)**end for**(10)**return***results*

Third, we encode the terms extracted from posts with codes of terminology from BV. The algorithm for encoding the terms is in *TermEncoder*(). Generally, a terminology code is regarded as a concept code from various knowledge sources, including the UMLS Metathesaurus, SNOMED CT, and International Classification of Diseases 10th Revision. Thus, a terminology code represents the unique identity of a concept in different knowledge bases. Semantic relationships are easily identified in knowledge sources on the basis of IDs. Here, an encoding template is considered as a quadruple form (terminology ID, semantic type, knowledge source, and preferred name), which contains all of the necessary information of the extracted terms. After each term is identified with a specific domain in *DomainDiscovery*(), the terms are encoded mainly by using a template. The use of mixed domain terminology sets in the template helps encode special health-related descriptions, such as adverse drug events [27]. Then, in (7), we use a 2-tuple form (knowledge base, code) to describe the encoded codes for a specific medical term.

(7) encoded_term=(term,{(KB,code)})

For example, given the term “aspirin”, we encode it as (SNOMED CT, 387458008|aspirin|), which denotes that the term “aspirin” belongs to a domain in SNOMED CT encoded as 387458008 in accordance with (7). When encoding is completed, all terms are encoded and subsequently used to extract potential relationships among concepts from knowledge bases.

Next, we extract descriptive and semantic relationship items within the encoded text obtained in the previous step. Descriptive items refer to the basic structure of adjacent terms and their location relationships and are usually associated with the syntactic structure found on the basis of the results of MetaMap. Semantic relationship items are utilized to infer specialization or generalization relationships from UMLS. On the basis of the two types of items, we expand the text with both latent terms and the corresponding relationships found in *KB* as expressed in (8).

(8) expanded_text={terms}∪{latent_terms∊KB}

Finally, to facilitate the expansion of the explicit knowledge of posts, we extract descriptive items that describe expression patterns on the basis of term-encoded texts. Afterward, many descriptive items are discovered and re-encoded in accordance with their order of appearance in the text. The output in this step is item-encoded texts. The resulted encoded texts can be mapped to codes in UMLS in the output process. Labeling existing relationships as an item-encoded text reveals semantic meanings within the narrative text. We then discover the latent knowledge related to the extracted knowledge from the items. The latent knowledge discovery algorithm is presented in Algorithm 3.

**Algorithm 3** Latent knowledge discovery algorithm**Input:** knowledge graphs of explicit terms within text, the number of latent layers
**Output:** knowledge graphs of latent terms
(1)**Let***rels*^0^←{(*s*^0^, *r*^0^, *t*^0^)} from knowledge graphs within text(2)**Let***rels^z^*←an empty list of (*s*, *r*, *t*), and *z* is the number of latent layers(3)**for each** (*s*^0^, *r*^0^, *t*^0^) ∊ *rels*^0^ **do**(4)    **if** normalized *s*^0^ ∊ UMLS **then**(5)        **Let** *d* ← distance between *s*^0^ and *t* ∊ UMLS concepts(6)        *rels^z^*→{*(s*^0^,*r*,*t*)*^z^*|∀*r* ∊ UMLS relations, ∀*t* ∊ UMLS concepts, min(*d*) = *z*}(7)    **end if**(8)**end for**(9)**return***rels^z^*


By using Algorithm 3, we extract various implicit relationships in the framework on the basis of the limited explicit knowledge found in the posts. By reorganizing explicit and implicit knowledge relationships among the extracted terms within the text, the system provides knowledge-based services to online patients on the basis of their online posts. The results of Algorithm 3 can establish knowledge graphs for knowledge reasoning to improve the health management of online patients. This method provides novel techniques for integrating domain knowledge support in OHCs. We next discuss the evaluation metrics to be used to examine the performance of our proposed method. We also discuss the improvement in promoting knowledge-based support for online patients.

### 2.3. Evaluation Metrics

Information systems in OHCs must use reliable evaluation metrics to further improve the knowledge-based services provided by OHCs when the proposed method is applied to analyze online posts.

#### 2.3.1. Explicit Knowledge Rate

Determining whether a post is suitable for knowledge support is essential for knowledge support systems in OHCs. The Explicit Knowledge Rate (*EKR*) is used to describe the richness of medical knowledge directly found in a post (*k*).

(9) $$EKR=d_{k}=\frac{n_{k}}{n_{raw}}$$

In (1), *d_k_* stands for the *EKR* of post *k*, *n_k_* is the number of medical concepts in the encoded text, and *n_raw_* denotes the number of all terms in the narrative text before they are encoded. This metric is useful in determining essential posts among the massive number of unlabeled posts in an OHC [28]. The *EKR* follows strict relationships in the expert knowledge of UMLS to ensure the correctness and relevance of extracted medical terms. Therefore, this metric improves the evaluation of the degree of knowledge support provided by online posts.

#### 2.3.2. Latent Knowledge Rate

Latent knowledge, in contrast to the explicit knowledge contained by a post, is extracted from DKSF in accordance with our method. Latent Knowledge Rate (*LKR*) measures the ratio of latent knowledge contained by a post by evaluating the implicit knowledge extracted from a post. LKR is calculated as follows:

(10) $$LKR=r_{p}=\frac{k_{r}-k_{d}}{k_{d}},$$

where *r_p_* denotes a *LKR* value, *k_r_* denotes the number of medical concepts in our proposed method associated with *KB*, and *k_d_* denotes the number of explicit concepts in a post. *K_r_* is calculated as

(11) $$K_{r}=\sum_{a=1}^{n_{k}}\sum_{i=1}^{N_{u}}rel(a,i)\sum_{j=1}^{N_{u}-1}rel(i,j)\ldots \sum_{x=1}^{N_{u}-m}rel(x-1,x),$$

where *N_u_* denotes the number of concepts in the UMLS Metathesaurus; *a* denotes the *a*-th concepts found in posts; *i*, *j*, and *x* denotes the *i*-th, *j*-th, and *x*-th concepts in the UMLS, respectively; *m* denotes the number of searching layers in the KH; and *rel*(*i*, *j*) indicates whether semantic relations exist between the *i*-th and *j*-th concepts. If *rel*(*i*, *j*) ≥ 1, certain relationships exist between two concepts; otherwise, the two concepts are unrelated.

*LKR* is the second metric for evaluating the extent of implicit knowledge extraction from posts. The number of searching layers (*m*) in the KH largely influences the performance of *LKR* in the KI-TM method because increasing the number of layers causes implicit knowledge to rapidly increase.

#### 2.3.3. Knowledge Correlation Rate

The evaluation of the correctness of the extracted knowledge is extremely important in existing information extraction methods. In general, one evaluates the correctness of extraction by using a gold standard. Unfortunately, no such gold standard exists in the real word. However, the UMLS is a massive expert knowledge source that can be regarded as a gold standard. Thus, we propose the Knowledge Correlation Rate (*KCR*) as a novel metric for evaluating extracted knowledge on the basis of the expert knowledge provided by the UMLS.

Supposing that *KG*_0_ is the extracted knowledge graph and *KG_U_* is the normalized knowledge graph, we have

(12) $$KCR=\frac{KG_{0}}{KG_{U}}=\frac{n_{s}}{n},$$

where *KG* is defined as a group of entities {concept_relation}. *N_s_* is the available relations found in UMLS, and *n* is the total number of available relations inferred in accordance with the expert knowledge provided by the UMLS.

If all the relationships in *KG*_0_ are semantically found in *KG_U_*, the value should be 1. *KCR* provides a simple and reasonable metric for solving the correctness problem.

#### 2.3.4. Perplexity of KI-TM

To quantify the ability of the KI-TM method to predict post topics, we introduce the Perplexity of KI-TM (PK) for the evaluation of LDA-based models [29]. A low perplexity value indicates that the probability distribution is conducive for sample prediction. Given the KI-TM model *q*, it may be evaluated by asking how well it predicts separate test samples *x*_1_, *x*_2_, ..., *x_N_*, which are also drawn from *p*. The perplexity (*p_k_*) of model *q* is defined as

(13) $$Perplexity=PK=b^{-\frac{1}{N}\sum_{i=1}^{N}\log_{b}q(x_{i})}$$

where *b* is customarily 2. Superior models (*q*) of unknown distribution (*p*) will tend to assign high probabilities (*q*(*x_i_*)) to test events. Thus, they have low perplexity, that is, they are less surprised by the test sample.

In summary, *EKR* and *LKR* are used to evaluate the degree of knowledge discovery in the analysis of online posts. *PK* is used to evaluate the performance of the KI-TM model.

## 3. Results

### 3.1. Datasets

We collected a dataset containing 372,343 patient-reported posts from an OHC for analysis. In this dataset, posts contain narrative texts in English that are related to 671 different kinds of drugs. Given that the website’s terms of use prevents privacy disclosure, we only utilize public information, such as narrative texts and their captions in posts, for academic purposes. On the basis of this dataset, we use the proposed evaluation metrics to examine the performance of our method in processing online posts in OHCs and the advantages it provides for improving knowledge-based services in OHCs. Table 2 presents examples of the posts we used in our analysis. Note that, the contents of the posts we used may contain some spelling irregularities or erroneous contents, which is normal because the texts are written in human language. The analysis process should consider a process of standardization of terminology for the posts.

First, we examine the advantages of *EKR* and *LKR* in evaluating patient-generated posts to promote knowledge support in OHCs. The analysis of the narrative contents of posts in most OHCs is facilitated by keyword-based matching methods, which are easily integrated into existing OHC frameworks [30]. Here, we compare the performance of the proposed method with that of the keyword-based matching method.

Second, we use perplexity as an evaluation metric to evaluate the ability of our proposed KI-TM method to process the online posts. We compare our method with LDA-based methods [31,32] and discuss its ability to discover knowledge from posts.

### 3.2. Knowledge Support Provided by the Proposed Method

**Figure 4:**
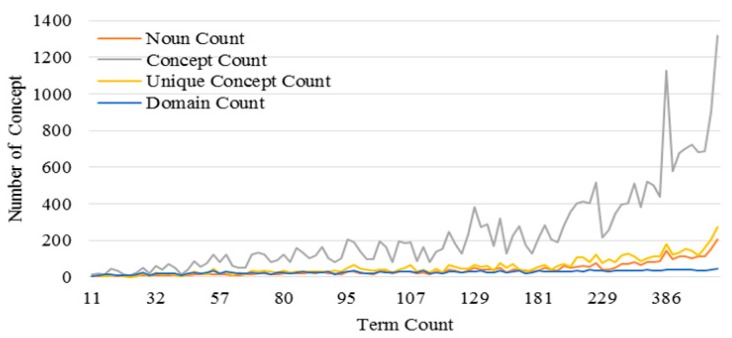
Numbers of different types of concepts extracted from posts with different lengths.

To examine the degree of knowledge support provided by our proposed method, we implement an experiment based on our dataset. First, we compare the changes in the *EKR* of the keyword- and UMLS-based methods with increasing post lengths. The keyword-based sample data yield a skewness of 3.25 and a kurtosis of 22.91, whereas the UMLS-based sample data yield a skewness of 1.80 and a kurtosis of 6.95. The reported *P*-values of the two-sample test (Mann–Whitney Test, α = 0.05, *p* < 0.001) of the two methods indicate that the knowledge extracted from posts by using our method is more concise but more useful than that by using the keyword method. Second, we examine the changes in the number of concepts extracted from the UMLS versus the number of terms within posts. The results are shown in Figure 4. One can find that all changes to the number of concepts in the different cases increase while the number of extracted terms increases. However, more duplicated concepts can also be found among the terms. If we remove these duplicated concepts, the increasing rate of the unique concept count found among the terms will rapidly decrease. In summary, the results in Figure 4 illustrate the fact that there are many redundant concepts in the posts that may affect our analysis, although the redundancy sometimes turns out to be a useful metric, such as using the frequencies of duplicated concepts as the weights for concepts.

**Figure 5:**
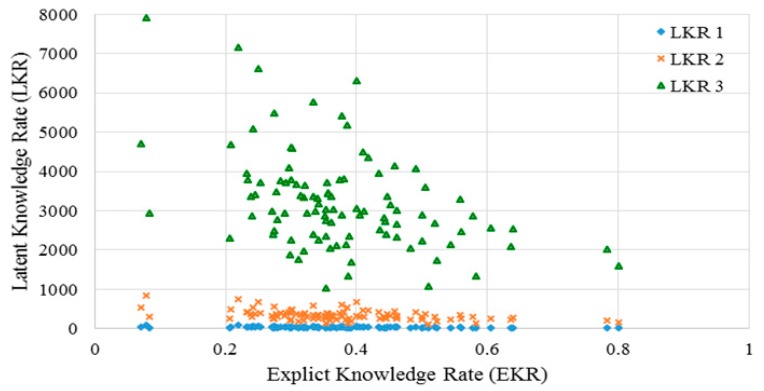
Change in latent knowledge rates (*m* = 1, 2, 3) over explicit knowledge rate.

Afterwards we examined the correlation between *EKR* and *LKR* of the posts used in the analysis. The results are shown in Figure 5. The changes of *LKR*s (with *m* = 1, 2, 3) versus different *EKR*s ranging in [0, 1] show great difference. The output of *LKR* for the same *EKR* may be different when using a different value of *m*. The results in Figure 5 illustrate that modifying the parameter *m* in the *LKR* can expand implicit knowledge outside the posts. In addition, the parameter *m* can be adjusted to meet different requirements of the expansion of expert knowledge found in the posts. According to the results, the UMLS based method can outperform the traditional methods, extracting more semantically implicit knowledge from the narrative text of posts by introducing the *LKR* metric in our study. The findings provide the basis for our KI-TM method, the performance of which is examined in the following section.

Therefore, utilizing posts by using existing external knowledge sources in medical domains is more practical than extracting explicit knowledge from posts on the basis of the determined number of health-related concepts in an OHC.

### 3.3. Performance of the KI-TM Method

**Figure 6:**
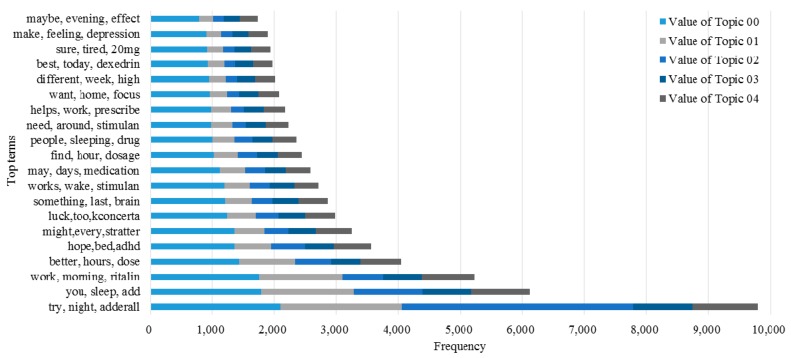
Comparison of frequency values of five topics with top five terms by using original posts.

**Figure 7:**
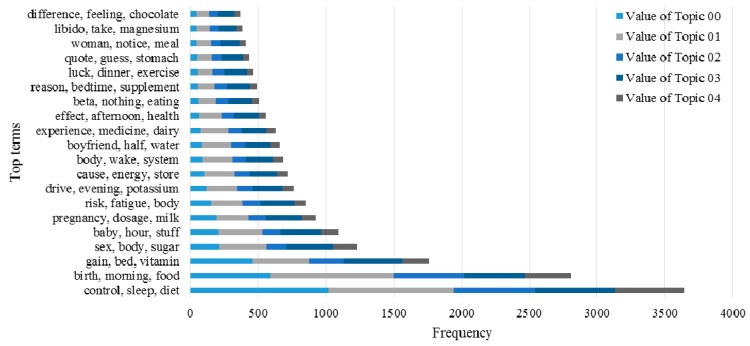
Comparison of frequency values of five topics with top five terms by using Knowledge-Involved Topic Modeling (KI-TM) with Latent Knowledge Rate (*LKR*) (*m* = 1).

**Figure 8:**
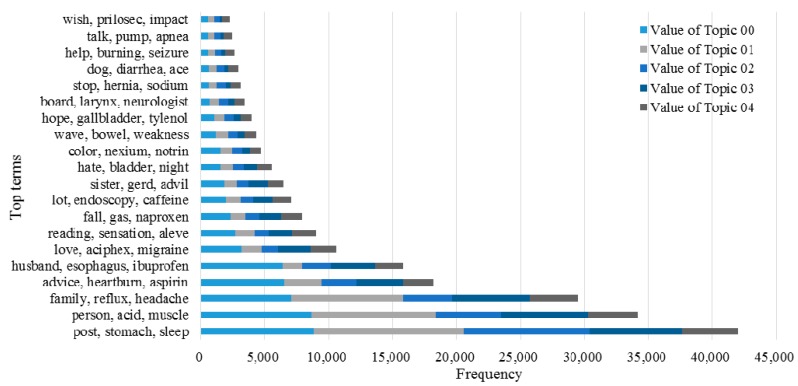
Comparison of frequency values of five topics with top five terms by using KI-TM with *LKR* (*m* = 2).

Here, we use *LKR* with different *m* values to examine the ability of the KI-TM method to provide meaningful healthcare-related knowledge support to online patients. We process the same posts in three cases. Figure 6 shows the results of LDA in the first case, which uses original posts. Figure 7 shows the results of LDA in the second case, which uses *LKR* (*m* = 1). The results show that the knowledge extraction from posts is extended by searching an additional layer in the knowledge network of UMLS. Figure 8 shows the results of LDA in the third case, which uses *LKR* (*m* = 2). In this case, knowledge extraction is extended by searching two additional layers in the knowledge network of UMLS. Figure 6, Figure 7 and Figure 8 illustrate the difference in frequency and top terms of topics obtained under different *m* values of *LKR*. The top terms shown in Figure 8 are more meaningful than those shown in Figure 6 and Figure 7. To summarize, using *LKR* with higher *m* in LDA methods increases the accuracy of extracting healthcare-related terms for determined topics in terms of DKS.

**Figure 9:**
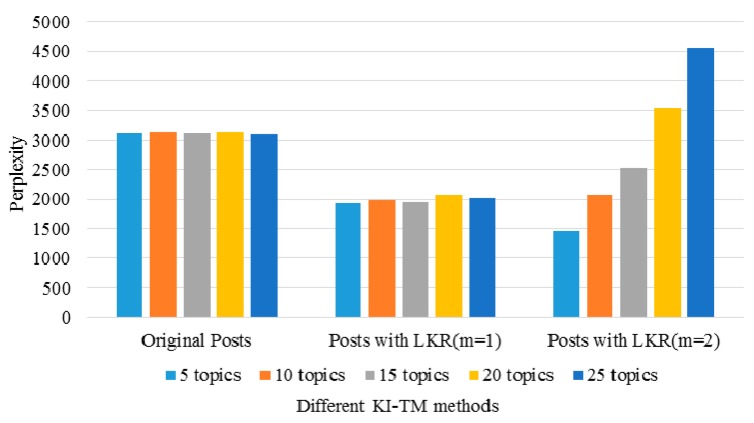
Comparison of the perplexity values of different Latent Dirichlet Allocation (LDA) based methods.

We also examine the performance of the *PK* method in these cases. The results are shown in Figure 9. In general, a low perplexity in (13) indicates a sure and superior model. As shown in Figure 9, the perplexity using posts with *LKR* (*m* = 1) is lower than that using original posts. The method using original posts is to use traditional methods without domain knowledge involved. The perplexity obtained by using original posts and *LKR* (*m* = 1) remains stable when the number of topics continuously increases. The perplexity obtained by using posts with *LKR* (*m* = 2) increases with the number of topics, although when using less than 20 topics, the perplexities are lower than that obtained by using the original posts. As we can see, involving *LKR* in LDA based methods can ultimately result in a good model, as shown in Figure 9. With the increasing *m*, the perplexity using less than 20 topics tends to decrease continually and thus provide better results than those without using domain knowledge. The essential reason of this change is that the introduction of the LKR changes the outputs of feature selections within LDA methods. Other parameters may also affect these results such as the 25 topics we assigned in the third experiment in Figure 9. For example, when analyzing posts using *m* = 2 and 25 topics, we can see the perplexity is larger than that using the original post. In this case, a novel selection technique of the results may be necessary to determine the proper number of topics we need when implementing such techniques. Therefore, the changes in perplexity in these cases indicate that our KI-TM method with *LKR* produces more interpretable topics and surer models than the traditional LDA-based methods.

## 4. Discussion

Our method aims to improve the knowledge support in OHCs to thereby improve the ability of online patients to self-manage their conditions. It applies domain knowledge in the medical field on the basis of the UMLS. Posts written by online patients sharing their experiences with other patients with similar conditions provide various valuable medical data, which can be utilized through the application of the proposed method. We reconstruct post content by using the KI-TM method to extract and expand medical knowledge contained by a post. Machine-learning techniques are easily implemented as the first step in the analysis of patient-reported posts given the dependence of these techniques on effective feature extraction from the posts [33].

The advantage of our approach in improving decision making for online patients is obvious according to the experimental results. Comparing the performance of the keyword-matching method with that of the UMLS-based method indicates that knowledge extracted from posts through the proposed method contributes to the complete understanding of knowledge in medical domains because of the full involvement of massive domain knowledge provided by the UMLS Metathesaurus illustrated in Figure 1, and as the results show in Figure 4 and Figure 5. Traditionally, an OHC uses keyword-matching and other metadata-based methods to identify the topics of the posts for decision-making. Then the OHC tries to recommend relevant posts to online patients. However, the proposed UMLS based approach focuses on introducing healthcare related concepts and their semantic relationships to enhance the understanding of posts in medical and healthcare perspectives, as described in Figure 2. Our novel approach leads to expanded expert knowledge found in such posts and thus decision support for online patients will become more reliable than using traditional methods. The novel approach also affects initial input of applying machine-learning techniques in the future, such as text clustering and text classification techniques, because most of them need clear inputs before implementing their algorithms for good results, as illustrated in Figure 6, Figure 7 and Figure 8. Wrong and inaccurate inputs for the machine learning techniques may result in misleading and confusing results. Systems in OHCs can thus provide enhanced knowledge-based services to online patients in accordance with medical knowledge. Moreover, the extraction of medical information from a health-related post through the keyword-based matching method is less useful and even misleading for OHCs because the keywords may include nonmedical information. Keyword-matching methods fail to extract expert knowledge that could help online patients. In our study, we also develop four key metrics, *EKR*, *LKR*, *KCR*, and *PK*, to evaluate the expert knowledge found in the posts so the knowledge can improve the services provided by information systems in OHCs. In fact, the information systems in the OHCs are essential to help facilitate our method in the whole process of enhancing the services for online patients. The *EKR* enhances the ability of monitoring the extracted explicit knowledge found in the posts while the *LKR* focuses on monitoring the expanding rate of implicit knowledge, the experimental results of which are shown in Figure 4 and Figure 5. The *KCR* is novel and practical for evaluating the reliability of extracted semantic relationships. The *PK* is used in our KI-TM method as a common metric to evaluate the performance of LDA based methods. Our experimental results indicate that relevant medical concepts, together with implicit concepts and relationships in the healthcare field, can be extracted from posts through the proposed method, which helps improve knowledge discovery from the posts in OHCs. The results of perplexity confirm that our method produces more interpretable topics and surer models in terms of knowledge support for online patients than keyword-based methods.

However, our work has several limitations. First, although we compared the proposed method with the keyword-matching method, which is widely used in practical OHCs, different OHCs may use different text-mining techniques and may consider numerous nonmedical factors, such as geographical information, to improve their knowledge support services. Second, our proposed method does not aim to replace typical machine-learning techniques [34]. Instead, we use the proposed method during the initial stages of machine-learning techniques, such as the removal of useless terms, when subjecting posts to text classification. Third, UMLS and SNOMED CT are licensed products that require licenses for practical use. Therefore, the license requirement may limit the use of our method in OHCs. However, if organizations are willing to develop open licenses for public use, the utilization of domain knowledge will considerably promote the development of OHCs. Lastly, the collected dataset is relevant to drug use and written in natural language. Hence, our experimental results may not have a reference value for some specific OHCs, such as PatientsLikeMe, which is a website using patient’s journals and charts to quantify health data. In this case, analyzing structured online health reports will still involve the support of medical expert knowledge because it could reduce the errors of mining methods and will guide online patients by providing trusted knowledge-based services.

## 5. Conclusions

We proposed a comprehensive knowledge discovery method that utilizes the UMLS and its core subset SNOMED CT to improve the knowledge-based services provided by OHCs. First, we proposed the analytical process. We then proposed DKFS, which provides a reference for designers of OHCs to improve their services to online patients. Second, we illustrated the use of medical concepts and their relationships based on domain knowledge to provide suggestions for improving OHCs. Third, we used four metrics to prove that the proposed methods can effectively discover knowledge from health-related posts. Finally, our experimental perplexity results indicated that the novel KI-TM method is suitable for producing highly interpretable topics and topics that are appropriate for the analysis of patient-reported posts. Our novel framework for OHCs, the KI-TM method, and effective evaluation metrics contribute to establishing an intelligent OHC for the effective healthcare management of patients in the future.

## Figures and Tables

**Table 1:** Element-mapping relationships between the UMLS Metathesaurus and our Domain-Knowledge Support Framework (DKSF).

Element Type of UMLS	Element Type of Our DKFS
Concept	Term
Concept names	Term names (standardized terms)
Relationships	Concept relationships
Attribute	Concept attribute (part-of-speech)
Source vocabularies	Domains (medical meanings)
String identifiers	Entity in narrative text
Lexical identifiers	Semantic relationships
Knowledge hierarchy	Knowledge hierarchy

**Table 2:** Example of posts in online health communities.

Name of Drug	Caption of Online Post	Main Body of Online Post
Atenolol	Low Libido	Wow..........is atenolol the answer? Bookish, I hope you get this resolved.... Sincerely, Oleander.
Diovan	Stopped Diovan—Hurrah!!	Hello, I take Diovan. I missed why you wanted to get off it? Bad side effects?
Tazorac	Should I Give Retin-A Micro the Boot	Tazorac is basically the same thing as Retin A accept it’s supposed to be more potent.
Trazodone	Generic Amb ien!?	trazodone—nonaddictive, no grogginess and something that I’d suggest to anyone.
Wellbutrin	I am Going to Quit Smoking Soon....but I have Panic Disorder	Wellbutrin really worked for me. I wish I had tried it years ago.
